# Supplementary material for: Giant nonlinear Hall and wireless rectification effects at room temperature in the elemental semiconductor tellurium
Source: Nat Commun. 2024 Jun 29;15:5513. doi: 10.1038/s41467-024-49706-y (PMC11217359; doi:10.1038/s41467-024-49706-y)
Supplement: Supplementary file 1 — Supplementary Information [file 41467_2024_49706_MOESM1_ESM.pdf]

<u>**Supplementary Information for:**</u>

Giant nonlinear Hall and wireless rectification effects at room temperature in the elemental semiconductor tellurium

Bin Cheng[1,2,3#], Yang Gao[1,2#], Zhi Zheng[1,2,3#], Shuhang Chen[4], Zheng Liu[2], Ling Zhang[1,2,3], Qi Zhu[4], Hui Li[5], Lin Li[1,2,3]*, Changgan Zeng[1,2,3]*

[1]*CAS Key Laboratory of Strongly-Coupled Quantum Matter Physics, and Department of Physics, University of Science and Technology of China, Hefei, Anhui 230026, China*

[2]*International Center for Quantum Design of Functional Materials (ICQD), Hefei National Research Center for Physical Sciences at the Microscale, University of Science and Technology of China, Hefei, Anhui 230026, China*

[3]*Hefei National Laboratory, University of Science and Technology of China, Hefei, Anhui 230088, China*

[4]*Department of Electronic Engineering and Information Science, University of Science and Technology of China, Hefei, Anhui 230026, China*

[5]*Institutes of Physical Science and Information Technology, Anhui University, Hefei, Anhui 230601, China*

# These authors contributed equally.

* Correspondence and requests for materials should be addressed to C. Z. (cgzeng@ustc.edu.cn), and L. L. (lilin@ustc.edu.cn).

Supplementary Note 1: Symmetry analysis

To understand the nonlinear Hall signal observed in experiments, we start from the symmetry analysis for the bulk sample. As schematically shown in Supplementary Fig. 1a, Te has a highly anisotropic crystal structure that belongs to the D_3 point group. The atoms form helical chains along the c-axis by covalent bonding, while neighboring chains are bonded into a hexagonal lattice in the ab plane via van der Waals forces. There are only two types of symmetric operations in Te crystal, i.e., a

three-fold rotation C_3 about the c-axis and two-fold rotation C_2 within the ab plane (see Supplementary Fig. 1b). The inversion symmetry is already broken in such a material.

Generally, the nonlinear conductivity can be defined as:

$$\mathbf{J}_\mathrm{i} = \chi_\mathrm{ijk}\, \mathbf{E}_\mathrm{j}\mathbf{E}_\mathrm{k} \tag{S1}$$

The coefficient χ_ijk is then a rank-three tensor.

For bulk Te with the D_3 point group symmetry, the nonlinear susceptibility χ_ijk can be expressed as[1]:

$$\chi_\mathrm{ijk} = \begin{pmatrix} \chi_\mathrm{xxx} & 0 & \chi_\mathrm{xyy} & 0 & \chi_\mathrm{xyz} & 0 \\ 0 & \chi_\mathrm{yyx} & 0 & \chi_\mathrm{yzx} & 0 & 0 \\ 0 & 0 & 0 & 0 & 0 & 0 \end{pmatrix}$$

$$= \begin{pmatrix} \chi_\mathrm{xxx} & 0 & -\chi_\mathrm{xxx} & 0 & \chi_\mathrm{xyz} & 0 \\ 0 & -\chi_\mathrm{xxx} & 0 & -\chi_\mathrm{xyz} & 0 & 0 \\ 0 & 0 & 0 & 0 & 0 & 0 \end{pmatrix} \tag{S2}$$

That is, χ_ijk has only two independent non-zero elements, i.e., χ_xxx and χ_xyz. Here, the coordinates x, z are chosen along the principal axis of the crystal, i.e., a-axis, and c-axis, respectively, while y is perpendicular to the ac plane. The electric field used in our experiments can be expressed as:

$$\mathbf{E} = (E_\mathrm{x}, 0, E_\mathrm{z}) \tag{S3}$$

The resulting second-order current density is depicted as follows:

$$\mathbf{J}^{(2)} = \begin{pmatrix} \mathbf{J}_\mathrm{x} \\ \mathbf{J}_\mathrm{y} \\ \mathbf{J}_\mathrm{z} \end{pmatrix} = \begin{pmatrix} \chi_\mathrm{xxx} E_\mathrm{x} E_\mathrm{x} \\ 2\chi_\mathrm{yzx} E_\mathrm{z} E_\mathrm{x} \\ 0 \end{pmatrix} \tag{S4}$$

Therefore, no second-order Hall current can develop along the a- and c-axis in pristine Te crystal.

I. C_2 symmetry breaking

In this case, we assume that the C_2 symmetry is lifted while the C_3 symmetry is reserved. The corresponding χ_ijk is then described as:

$$\chi_{\mathrm{ijk}}=\begin{pmatrix}\chi_{\mathrm{xxx}} & \chi_{\mathrm{xxy}} & \chi_{\mathrm{xyy}} & \chi_{\mathrm{xxz}} & \chi_{\mathrm{xyz}} & 0\\ \chi_{\mathrm{yxx}} & \chi_{\mathrm{yyx}} & \chi_{\mathrm{yyy}} & \chi_{\mathrm{yzx}} & \chi_{\mathrm{yyz}} & 0\\ \chi_{\mathrm{zxx}} & 0 & \chi_{\mathrm{zyy}} & 0 & 0 & \chi_{\mathrm{zzz}}\end{pmatrix}$$

$$=\begin{pmatrix}\chi_{\mathrm{xxx}} & -\chi_{\mathrm{yyy}} & -\chi_{\mathrm{xxx}} & -\chi_{\mathrm{xxx}} & \chi_{\mathrm{xyz}} & 0\\ -\chi_{\mathrm{yyy}} & -\chi_{\mathrm{xxx}} & \chi_{\mathrm{yyy}} & -\chi_{\mathrm{xyz}} & -\chi_{\mathrm{xxx}} & 0\\ \chi_{\mathrm{zxx}} & 0 & \chi_{\mathrm{zxx}} & 0 & 0 & \chi_{\mathrm{zzz}}\end{pmatrix} \tag{S5}$$

Driven by the electric field shown in Equation (S3), the resulting second-order current density is:

$$\mathbf{J}^{(2)}=\begin{pmatrix}\mathbf{J}_{\mathrm{x}}\\ \mathbf{J}_{\mathrm{y}}\\ \mathbf{J}_{\mathrm{z}}\end{pmatrix}=\begin{pmatrix}\chi_{\mathrm{xxx}}E_{\mathrm{x}}^2+2\chi_{\mathrm{xxz}}E_{\mathrm{x}}E_{\mathrm{z}}\\ \chi_{\mathrm{yxx}}E_{\mathrm{x}}^2+2\chi_{\mathrm{yzx}}E_{\mathrm{z}}E_{\mathrm{x}}\\ \chi_{\mathrm{zxx}}E_{\mathrm{x}}^2+\chi_{\mathrm{zzz}}E_{\mathrm{z}}^2\end{pmatrix} \tag{S6}$$

Such a second-order current is equivalent to an electric field $\mathbf{E}^{(2)}$:

$$\mathbf{E}^{(2)}=\rho\cdot\mathbf{J}^{(2)}=\begin{pmatrix}\rho_{\mathrm{x}} & 0 & 0\\ 0 & \rho_{\mathrm{y}} & 0\\ 0 & 0 & \rho_{\mathrm{z}}\end{pmatrix}\begin{pmatrix}\chi_{\mathrm{xxx}}E_{\mathrm{x}}^2+2\chi_{\mathrm{xxz}}E_{\mathrm{x}}E_{\mathrm{z}}\\ \chi_{\mathrm{yxx}}E_{\mathrm{x}}^2+2\chi_{\mathrm{yzx}}E_{\mathrm{z}}E_{\mathrm{x}}\\ \chi_{\mathrm{zxx}}E_{\mathrm{x}}^2+\chi_{\mathrm{zzz}}E_{\mathrm{z}}^2\end{pmatrix} \tag{S7}$$

We notice that second-order signals are allowed at the direction perpendicular to the ac plane. However, such an out-of-plane component will not be detected due to the fact that all the electrodes of our Te devices were fabricated within the ac plane, and is therefore neglected in the following analysis. The two components of $\mathbf{E}^{(2)}$ within the ac plane are:

$$\mathbf{E}_{\mathrm{xz}}^{(2)}=\begin{pmatrix}\mathbf{E}_{\mathrm{x}}^{(2)}\\ \mathbf{E}_{\mathrm{z}}^{(2)}\end{pmatrix}=\begin{pmatrix}\rho_{\mathrm{a}}(\chi_{\mathrm{xxx}}E_{\mathrm{x}}^2+2\chi_{\mathrm{xxz}}E_{\mathrm{x}}E_{\mathrm{z}})\\ \rho_{\mathrm{c}}(\chi_{\mathrm{zxx}}E_{\mathrm{x}}^2+\chi_{\mathrm{zzz}}E_{\mathrm{z}}^2)\end{pmatrix} \tag{S8}$$

For a current along arbitrary direction within the ac plane, the two in-plane first-order electric field components are:

$$\mathbf{E}_{\mathrm{xz}}^{(1)}=\begin{pmatrix}\mathbf{E}_{\mathrm{x}}\\ \mathbf{E}_{\mathrm{z}}\end{pmatrix}=\rho\cdot\mathbf{J}^{(1)}=\begin{pmatrix}\rho_{\mathrm{x}} & 0\\ 0 & \rho_{\mathrm{z}}\end{pmatrix}\cdot j\begin{pmatrix}-sin\theta\\ cos\theta\end{pmatrix}=j\begin{pmatrix}-\rho_{\mathrm{x}}sin\theta\\ \rho_{\mathrm{z}}cos\theta\end{pmatrix} \tag{S9}$$

where j is the amplitude and θ is the angle between the directions of the current and the c-axis. Therefore, the as-generated transverse component of the second-order electric field can be expressed as:

$$E_{\perp}^{(2)}=E_{\mathrm{x}}^{(2)}\cos\theta+E_{\mathrm{z}}^{(2)}\sin\theta$$

$$=\rho_{\mathrm{x}}^3j^2\sin\theta[\chi_{\mathrm{xxx}}\sin\theta\cos\theta+r\chi_{\mathrm{zxx}}\sin^2\theta+(2r\chi_{\mathrm{xxx}}+r^3\chi_{\mathrm{zzz}})\cos^2\theta] \tag{S10}$$

Here, $r = \frac{\rho_c}{\rho_a}$ is the in-plane resistance anisotropy that can be extracted from the angular dependent resistance data (as typically shown in Fig. 1c).

As shown in Fig. 1e, the $V_{xy}^{2\omega}$ versus θ data measured in device #D1 can be well fitted by Equation (S10).

Ⅱ. *C*3 symmetry breaking

In this case, we assume that the C_3 symmetry is lifted while the C_2 symmetry is reserved. The corresponding χ_{ijk} is then described as:

$$\chi_{ijk} = \begin{pmatrix} \chi_{xxx} & 0 & \chi_{xyy} & 0 & \chi_{xyz} & \chi_{xzz} \\ 0 & \chi_{yyx} & 0 & \chi_{yzx} & 0 & 0 \\ 0 & \chi_{zxy} & 0 & \chi_{zzx} & 0 & 0 \end{pmatrix} \tag{S11}$$

Driven by the electric field shown in Equation (S3), the resulting second-order current density is:

$$\mathbf{J}_{xz}^{(2)} = \begin{pmatrix} \mathbf{J}_x^{(2)} \\ \mathbf{J}_z^{(2)} \end{pmatrix} = \begin{pmatrix} \chi_{xxx}E_x^2 + \chi_{xzz}E_z^2 \\ 2\chi_{zzx}E_zE_x \end{pmatrix} \tag{S12}$$

It is obvious that second-order Hall current cannot develop along the c-axis with the electric field applied along the a-axis, which contradicts with our experimental observations.

Ⅲ. *C*3 and *C*2 symmetries both breaking

In this case, the point group is reduced to C_1, and the corresponding χ_{ijk} is then described as:

$$\chi_{ijk} = \begin{pmatrix} \chi_{xxx} & \chi_{xxy} & \chi_{xyy} & \chi_{xxz} & \chi_{xyz} & \chi_{xzz} \\ \chi_{yxx} & \chi_{yyx} & \chi_{yyy} & \chi_{yzx} & \chi_{yyz} & \chi_{yzz} \\ \chi_{zxx} & \chi_{zxy} & \chi_{zyy} & \chi_{zzx} & \chi_{zzy} & \chi_{zzz} \end{pmatrix} \tag{S13}$$

The as-generated transverse component of the second-order electric field can be expressed as:

$$E_{\perp}^{(2)} = E_x^{(2)}\cos\theta + E_z^{(2)}\sin\theta = \rho_a^3 j^2[\chi_{zxx}r\sin^3\theta + \chi_{xzz}r^2\cos^3\theta + (\chi_{xxx} - 2\chi_{zzx}r^2)\sin^2\theta\cos\theta + (\chi_{zzz}r^3 - 2\chi_{xxz}r)\sin\theta\cos^2\theta)] \tag{S14}$$

Similarly, we can write the equation for the longitudinal component as:

$$E_{\parallel}^{(2)} = E_z^{(2)}\cos\theta - E_x^{(2)}\sin\theta = \rho_a^3 j^2[-\chi_{xxx}\sin^3\theta + \chi_{zzz}r^3\cos^3\theta + r(2\chi_{xxz} + \chi_{zxx})\sin^2\theta\cos\theta$$

$$-r^2(\chi_{xzz} + 2\chi_{zzx}) \sin\theta \cos^2\theta] \tag{S15}$$

To check their validity, we combined the Equations (S14) and (S15) into a single group and fitted the data for $V_{xy}^{2\omega}$ and $V_{xx}^{2\omega}$ simultaneously. As shown in Supplementary Fig. 9, both the Hall and longitudinal data can be well described.

Supplementary Note 2: Scaling equation for etching-modified NLHE

As mentioned in the main text, the presence of second-order signals in both the longitudinal and transverse directions with comparable amplitude indicates a dominant extrinsic mechanism in Te. This is because the scattering-related extrinsic mechanism does not adhere to the relaxation time approximation as the intrinsic BCD should[2,3]. To get further insight, scaling law analysis is then employed, aiming to quantitatively describe the weights of intrinsic and extrinsic contributions using a multi-parametric scaling relation. Generally speaking, there are two types of scatters in transport, i.e., the static impurity such as the lattice imperfection, and the dynamic impurity such as the phonon. According to the Matthiessen's rule, we have $\rho_{xx} = \rho_{xx0} + \rho_{xxT}$, where ρ_{xxT} and ρ_{xx0} are the resistivities from phonon scattering and static impurities, respectively. The scaling behavior can in principle be formulated with respect to each type of scatters.

Following previous reports, the scaling equation with respect to the static impurity for the second-harmonic nonlinear Hall signal can be expressed as[2]:

$$\frac{E_{xy}^{2\omega}}{(E_{xx}^{\omega})^2} = \frac{\chi_{yxx}}{\sigma} = \mathrm{C^{in}} + \mathrm{C_0^{sj}}\frac{\rho_{xx0}}{\rho_{xx}} + \mathrm{C_1^{sj}}\frac{\rho_{xxT}}{\rho_{xx}} + \mathrm{C_{00}^{sk,1}}\frac{\rho_{xx0}^2}{\rho_{xx}^2} + \mathrm{C_{01}^{sk,1}}\frac{\rho_{xx0}\rho_{xxT}}{\rho_{xx}^2} + \mathrm{C_{11}^{sk,1}}\frac{\rho_{xxT}^2}{\rho_{xx}^2} + \mathrm{C^{sk,2}}\frac{\rho_{xx0}}{\rho_{xx}^2}$$
$$= \mathrm{a} + \mathrm{b}\frac{1}{\rho_{xx}} + \mathrm{c}\frac{1}{\rho_{xx}^2}, \tag{S16}$$

The scaling parameters a, b, c are depicted as:

$$\mathrm{a} = \mathrm{C^{in}} + \mathrm{C_0^{sj}} + \mathrm{C_{00}^{sk,1}}$$
$$\mathrm{b} = \left(\mathrm{C_1^{sj}} - \mathrm{C_0^{sj}}\right)\rho_{xxT} - 2\mathrm{C_{00}^{sk,1}}\rho_{xxT}^2 + \mathrm{C_{01}^{sk,1}}\rho_{xxT} + \mathrm{C^{sk,2}}$$
$$\mathrm{c} = \mathrm{C_{00}^{sk,1}}\rho_{xxT}^2 - \mathrm{C_{01}^{sk,1}}\rho_{xxT}^2 + \mathrm{C_{11}^{sk,1}}\rho_{xxT}^2 - \mathrm{C^{sk,2}}\rho_{xxT}$$

Below we discuss the effect of surface etching on the NLHE. As the etching changes the surface roughness and the thickness, the residual resistivity ρ_{xx0} varies but ρ_{xxT} stays the same since the measuring temperature is kept fixed. The above scaling law shown in Equation (S16) then suggests

that the sign reversal of $V_{xy}^{2\omega}$ can occur when the parameters a, b and c have different signs. Specifically, in our experiments, we find that ρ_{xxT} is much smaller compared to ρ_{xx0}, which can be deduced from the temperature dependent resistivity data shown in Supplementary Fig. 3a. We then take $\rho_{xxT} \approx 0$, and reduce the scaling law to the following form:

$$\frac{E_{xy}^{2\omega}}{(E_{xx}^{\omega})^2} = a' + \frac{b'}{\rho_{xx}} \tag{S17}$$

where $a' = C^{in} + C_0^{sj} + C_{00}^{sk,1}$ and $b' = C^{sk,2}$. The opposite sign of a' and b' can naturally lead to a sign reversal of $V_{xy}^{2\omega}$ when the resistivity varies.

Supplementary Note 3: Scaling law analysis on the temperature dependent NLHE behaviors

When the temperature decreases, the residue resistivity is approximately constant and the phonon scattering decays. To better describe such behavior, we reformulate Equation (S16) as follows[4]:

$$\begin{aligned} \frac{E_{xy}^{2\omega}}{(E_{xx}^{\omega})^2} = \frac{\chi_{yxx}}{\sigma} &= \left[C^{sk,2}\sigma_0 + \left(C_{00}^{sk,1} + C_{11}^{sk,1} - C_{01}^{sk,1}\right)\right]\sigma_0^{-2}\sigma^2 \\ &+\left(C_0^{sj} + C_{01}^{sk,1} - 2C_{11}^{sk,1} - C_1^{sj}\right)\sigma_0^{-1}\sigma + \left(C^{in} + C_1^{sj} + C_{11}^{sk,1}\right) \\ &= A_0 + A_1\sigma + A_2\sigma^2 \end{aligned} \tag{S18}$$

where

$$\begin{aligned} A_0 &= C^{in} + C_1^{sj} + C_{11}^{sk,1} \\ A_1 &= \left(C_0^{sj} + C_{01}^{sk,1} - 2C_{11}^{sk,1} - C_1^{sj}\right)\sigma_0^{-1} \\ A_2 &= \left[C^{sk,2}\sigma_0 + \left(C_{00}^{sk,1} + C_{11}^{sk,1} - C_{01}^{sk,1}\right)\right]\sigma_0^{-2} \end{aligned}$$

Here, σ_0 is the zero-temperature conductivity. As presented in Supplementary Fig. 12a, the typical $\frac{E_{xy}^{2\omega}}{(E_{xx}^{\omega})^2}$ versus σ curve, which is extracted from the nonlinear Hall data measured at various temperatures in device #D1 (the same device in Fig. 1b), can be well fitted by Equation (S18). By using the yielded values of A_2, A_1 and A_0 (A_2= 1.226×10^5 μm^{3}Ω^{2}V^{-1}, A_1= -1.526×10^3 μm^{2}ΩV^{-1}, A_0= 4.765 μmV^{-1}), the temperature dependence of χ_{yxx} and its three components ($A_2\sigma^3$, $A_1\sigma^2$ and $A_0\sigma$) are further plotted in Supplementary Fig. 12b. It is clearly seen that contribution scaling as $A_1\sigma^2$ is

almost two times larger than the other two items, even though its sign is opposite with the final-obtained nonlinear Hall signal in this device. Therefore, the modification of competition between the contribution of $A_1\sigma^2$ and those of $A_2\sigma^3$ and $A_0\sigma$ may lead to the sign reversal of the measured NLHE, which is consistent with our etching experiments. Since the intrinsic contribution originated from BCD only affect A_0, the relatively large proportion of $A_1\sigma^2$ and $A_2\sigma^3$ further verifies that the scattering-related extrinsic mechanisms play a leading role in inducing NLHE in our Te devices. However, a more thorough analysis is quite challenging due to the presence of numerous disorder sources and their competitions. To date, twisted bilayer graphene is among the few systems that could separate the contributions from different types of scattering to some extent[4]. However, the corresponding analysis relies on the specifics of both the twisted bilayer graphene system and the scaling law analysis data, and is not applicable for our Te device.

Supplementary Note 4: Exclusion of other possible origins of NLHE in Te

1. **Diode effect.** Possible Schottky contacts between samples and electrodes may lead to the formation of an accidental diode, giving rise to rectification effect and high-order transport phenomena[5]. However, such origin can be readily ruled out in our experiments, since well-defined linear *I-V* curves were obtained between arbitrary two electrodes via two-terminal DC measurements in our Te devices, as typically shown in Supplementary Fig. 4b.

2. **Thermoelectric effect.** Non-uniform Joule heating under AC current can give rise to a second-harmonic voltage drop due to the Seebeck effect, and the magnitude of the voltage can be expressed as[7]:

   $$V^{2\omega} \propto \Delta T \propto (I^{\omega})^2 R \tag{S19}$$

   The uniformity of Joule heating normally originates from external asymmetries, such as contact resistance difference and asymmetric sample shapes[8]. However, such thermoelectric effect should dominate along the longitudinal direction rather than the transverse one. As for our experiments, the measured second-harmonic signals are, however, comparable for both directions. On the other hand, the experimentally observed anisotropic $V_{\mathrm{xx}}^{2\omega}$, which is found to be closely linked to the crystalline symmetry of Te (see Supplementary Fig. 9), cannot be explained by the thermoelectric

effect either. These two findings thus rule out the thermoelectric effect as the underlying origin for the observed second-harmonic signals in our experiments.

3. **Electrode misalignment.** In actual devices, the unavoidable misalignment between electrodes can lead to a mixing of longitudinal signal to the transverse component. Nevertheless, from the data presented in Supplementary Fig. 2a, the corresponding amplitude of the as-mixed Hall signal is very small when compared to the longitudinal one (only 4%). Consequently, the contribution of longitudinal-signal-induced mixing to the observed NLHE can be reasonably neglected, according that the amplitude of the measured second-harmonic Hall voltage is comparable to that of the longitudinal one.

4. **Asymmetric sample shape.** The generation of second harmonic signal can occur when carriers are scattered by the boundaries of a sample with asymmetric shape[6]. However, this effect largely depends on the specific asymmetry details of individual samples, and thus should result in distinct angular dependencies of NLHE among different Te devices. This is in contrast with our results shown in Fig. 1e and Supplementary Fig. 5, wherein nearly identical angular dependencies are seen for devices with notably different sample boundaries.

Supplementary Note 5: NLHE performance for as-grown Te samples with different thicknesses

Supplementary Figure 10 shows the measured second-harmonic Hall voltages for devices with different thicknesses, all of which clearly exhibit a quadratic dependence on the applied AC current. As the thickness increases, the magnitude decreases noticeably. For instance, the nonlinear Hall voltage for Te flake with a thickness of 24 nm is 0.21 mV (Supplementary Fig. 10a). While for the 131 nm thick sample, this value drops to about 0.003 mV, which is nearly two orders of magnitude smaller (Supplementary Fig. 10c). This result provides unequivocal evidence supporting the proposed surface effect.

Supplementary Figures:

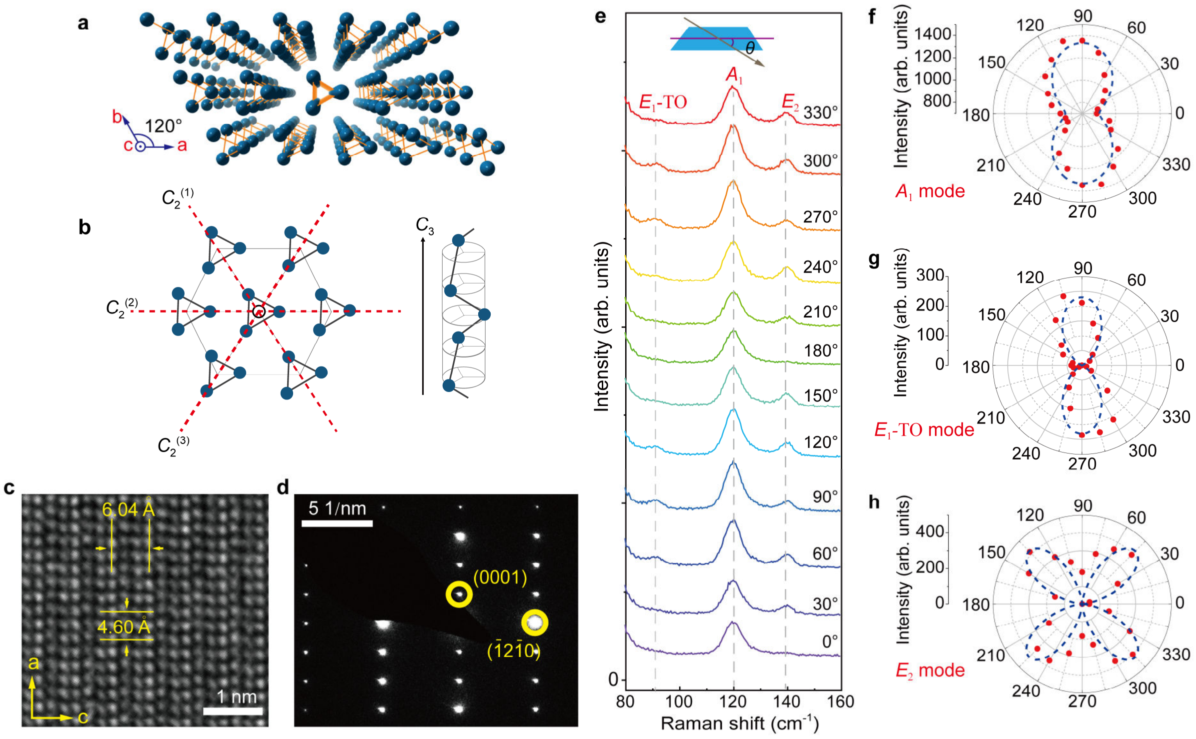

Supplementary Fig. 1 | Structural characterizations of Te thin flakes. a, Schematic of Te crystal structure. **b**, Top and perspective views of the crystal structure, in which the crystalline symmetries are marked. **c, d**, High-resolution transmission electron microscope and selective area electron diffraction pattern of the exposed surface of the as-grown Te thin flake. The surface is identified as the ac plane, with lattice parameters determined as a = 4.60 Å, c = 6.04 Å. **e**, Angle-resolved polarized Raman spectra for the as-grown Te flake. θ is defined as the angle between the polarization direction of laser and the long edge of the Te sample with trapezoidal shape (see the inset). There are three peaks visible, corresponding to E_1-TO, A_1 and E_2 modes, at 91 cm^{-1}, 120 cm^{-1} and 139 cm^{-1}, respectively. **f-h**, Polar plots of peak intensity for A_1, E_1-TO, and E_2 modes, respectively. The red dots are experimental data and the blue dashed lines are corresponding fitting curves. The fitting equations for these three modes are $I_{A_1} = |\mathrm{asin}^2\theta + \mathrm{bcos}^2\theta|^2$, $I_{E_1-\mathrm{TO}} = |\mathrm{csin}^2\theta|^2$ and $I_{E_2} = |2\mathrm{dsin}\theta\mathrm{cos}\theta|^2$, where a, b, c, and d are Raman tensor elements. This result further confirms the anisotropic crystallographic orientation of the as-grown trapezoidal-shaped Te flake, where the long edge represents the c-axis.

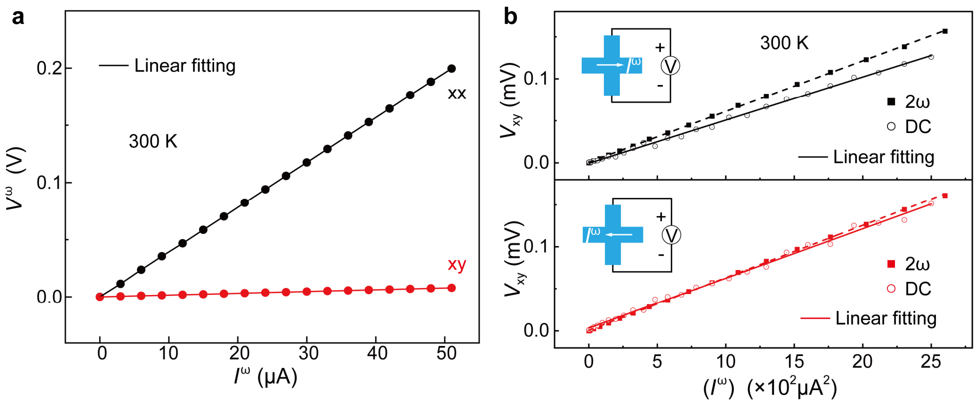

Supplementary Fig. 2 | Basic NLHE characterizations for device #D1. a, First-harmonic longitudinal and Hall voltage as functions of I^{ω}. **b**, Second-harmonic and DC Hall voltage as functions of $(I^{\omega})^2$, with the measuring configurations indicated in the insets. The solid lines in **a** and **b** are the linear fitting results. All the experimental data in Supplementary Figs. 2 to 12 were taken at 300 K with the AC current or oscillating electric field applied along the a-axis, unless otherwise noted.

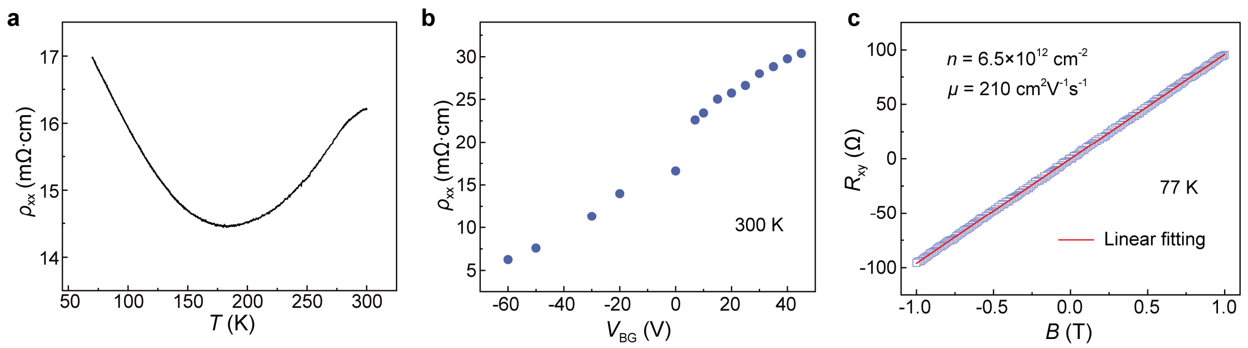

Supplementary Fig. 3 | First-harmonic transport characterizations for device #D1. a, Temperature dependent longitudinal resistivity. **b,** Longitudinal resistivity as a function of V_{BG}. **c,** Hall curve measured at 77 K and corresponding linear fitting result. The carrier density and mobility were calculated to be 6.5×10^{12} cm^{-2} and 210 $cm^2V^{-1}s^{-1}$, respectively.

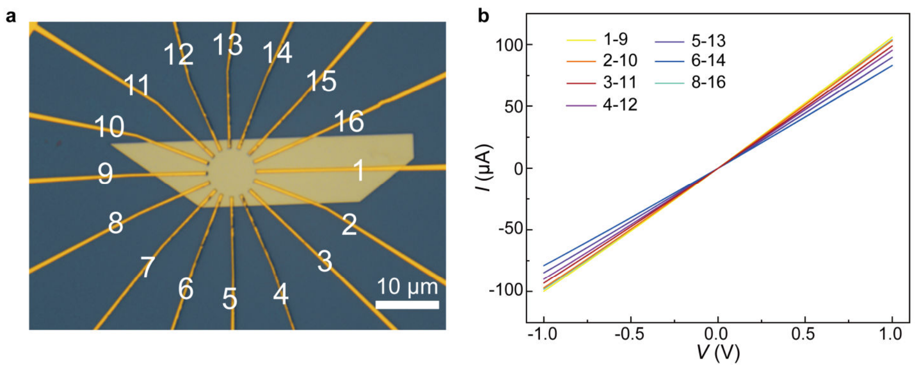

Supplementary Fig. 4 | DC current-voltage curves for device #D1. a, Optical image of the device #D1 with all the electrodes numbered. **b**, Two-terminal DC current-voltage curves between arbitrary two electrodes.

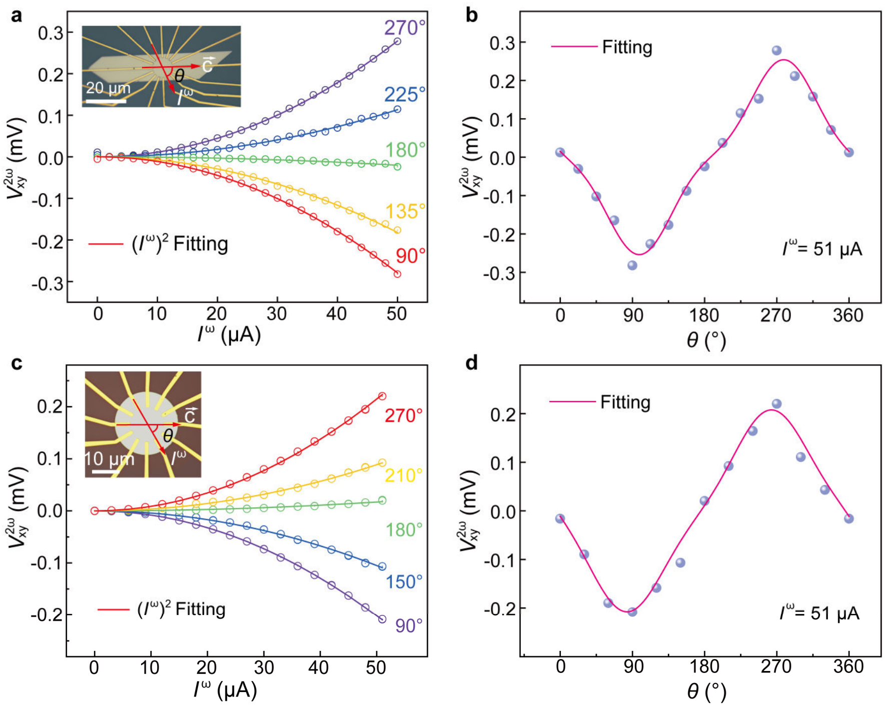

Supplementary Fig. 5 | Angular dependent NLHE in other two circular disc devices #D2 and #D3. a, c, $V_{xy}^{2\omega}$ as a function of I^{ω} at different angles for devices #D2 and #D3, respectively. The solid lines are the quadratic fitting results. Insets in **a** and **c**: optical images for the devices. Te flake used in device #D3 possesses a circular shape, which was achieved by etching the as-grown sample before electrode fabrication. **b, d,** Angular dependence of $V_{xy}^{2\omega}$ at I^{ω} = 51 μA and corresponding fitting result by using Equation (S14) for devices #D2 and #D3, respectively.

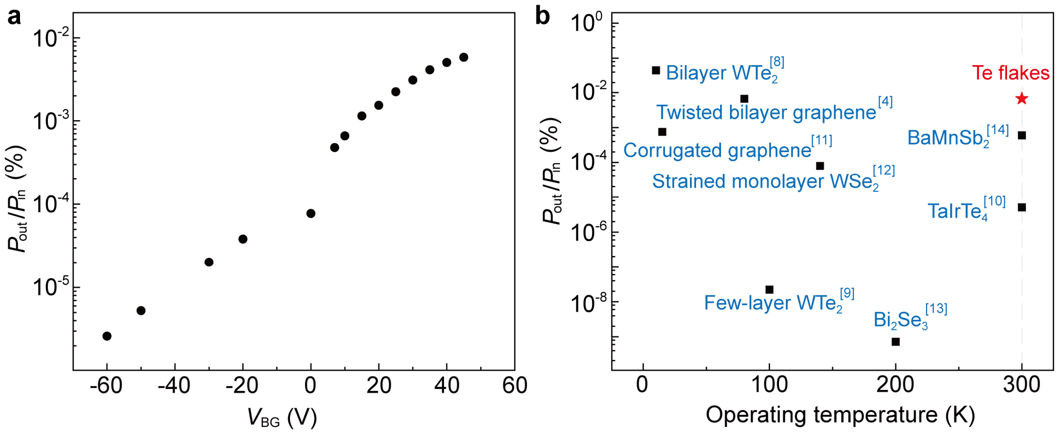

Supplementary Fig. 6 | Comparison of the power efficiency between different systems exhibiting NLHE. a, Power efficiency ($P_{\mathrm{out}}/P_{\mathrm{in}}$) as a function of V_{BG} for Te device #D1 (same as the one presented in Fig. 2). The power efficiency is estimated by using the equation[3]:$P_{\mathrm{out}}/P_{\mathrm{in}} = \left((V_{xy}^{2\omega})^2/R_{\mathrm{out}}\right)/((V_{xx}^{\omega})^2/R_{\mathrm{in}}) = (R_{\mathrm{in}}/R_{\mathrm{out}})\left(V_{xy}^{2\omega}/V_{xx}^{\omega}\right)^2$. **b,** Comparison of the NLHE performance among different systems by using $P_{\mathrm{out}}/P_{\mathrm{in}}$. Here the values of other systems are calculated using the data of $V_{xy}^{2\omega}$ and V_{xx}^{ω} from corresponding references. Since the precise values of R_{in} and R_{out} were usually not provided in most references, the ratio of $R_{\mathrm{in}}/R_{\mathrm{out}}$ was assumed to be 1 during the calculations. Such a simplified process is reasonable, since the reported in-plane resistance anisotropy for these systems are always small, e.g., around 1.14 for $BaMnSb_2$[14].

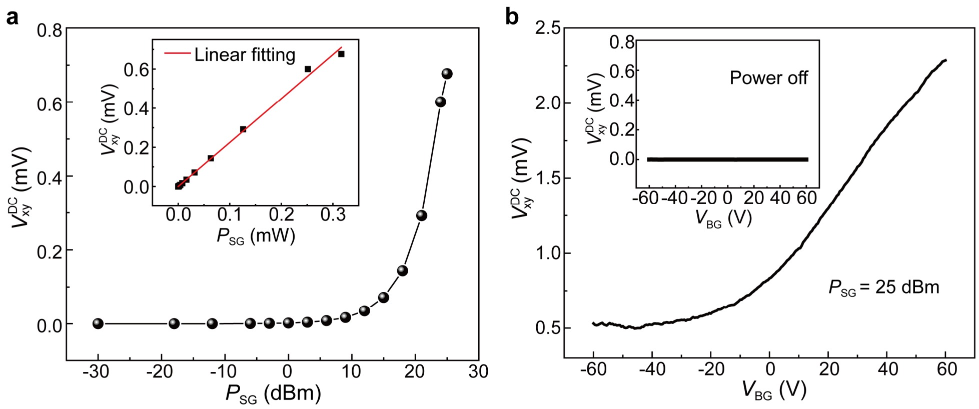

Supplementary Fig. 7 | Wireless rectification effect in cross-like device #C2. a, V_{xy}^{DC} as a function of P_{SG} with the RF frequency set to be 2.4 GHz. Inset: corresponding linear fitting result. **b,** V_{xy}^{DC} as a function of V_{BG} for P_{SG} =25 dBm. Inset: V_{BG} dependent V_{xy}^{DC} when the RF signal is off.

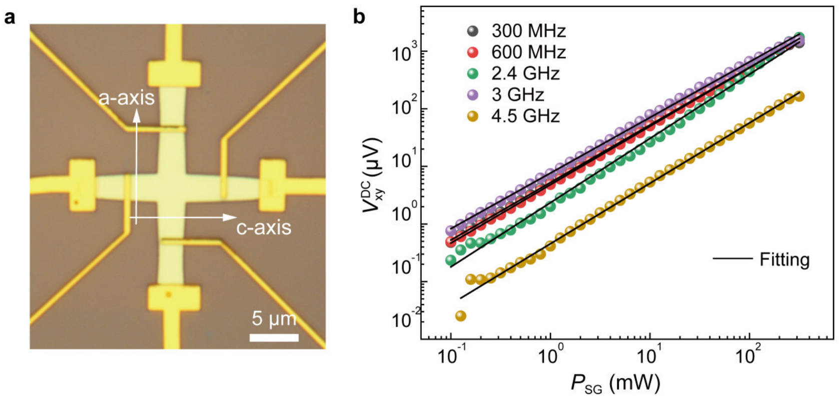

Supplementary Fig. 8 | Wireless rectification performances under different frequencies. a, Optical image of the cross-like device #C3. **b,** Logarithmic plots of $V_{\mathrm{xy}}^{\mathrm{DC}}$ versus P_{SG} curves. Several typical frequencies within the RF regime were adopted by using a log-periodic antenna. Solid lines are power-law fitting results using $V_{\mathrm{xy}}^{\mathrm{DC}} \propto (P_{\mathrm{SG}})^{\alpha}$. The extracted values of α are 1.00, 1.01, 1.02, 1.11, 0.96, 1.05 for 300 MHz, 600 MHz, 2.4 GHz, 3 GHz and 4.5 GHz, respectively.

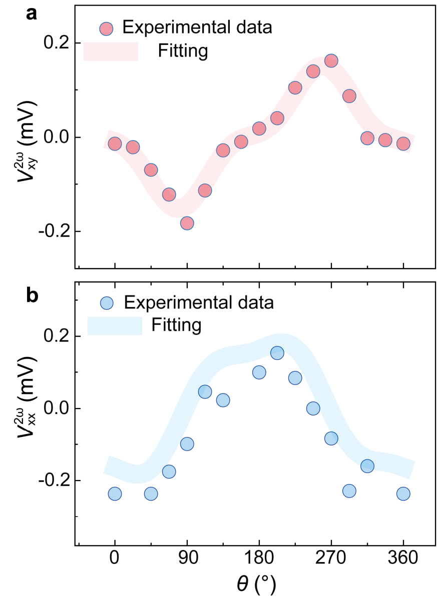

Supplementary Fig. 9 | Fitting results for angular dependent data. Angular dependence of (**a**) $V_{xy}^{2\omega}$ and (**b**) $V_{xx}^{2\omega}$ at I^{ω} = 51 μA for device #D1. The thick solid lines are the fitting curves, which were obtained by combining Equations (S14) and (S15) into a single group and then fitting the data for $V_{xy}^{2\omega}$ and $V_{xx}^{2\omega}$ simultaneously.

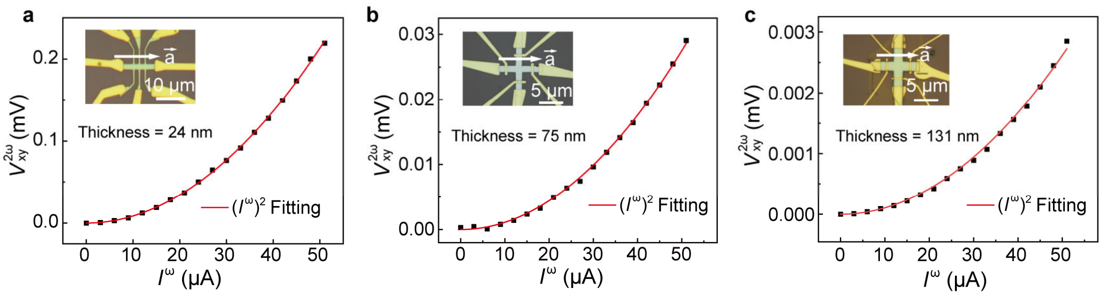

Supplementary Fig. 10 | NLHE in Te devices with different thicknesses. a-c, Second-harmonic Hall voltage as a function of the AC current for devices with three typical thickness. The red lines are corresponding $(I^{\omega})^2$ fitting curves. Insets in **a-c**: optical images of the devices.

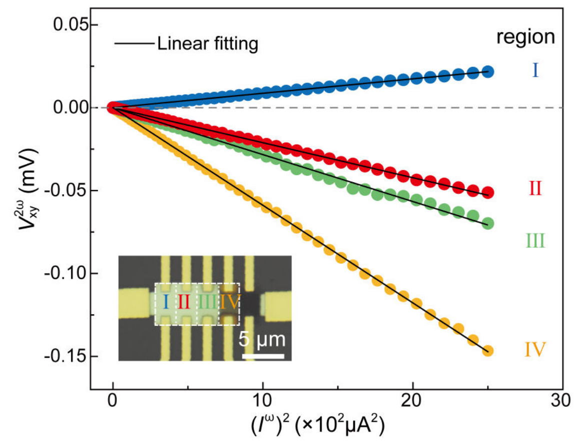

Supplementary Fig. 11 | Etching effect in another Hall-bar device #H2. $V_{xy}^{2\omega}$ versus $(I^{\omega})^2$ curves and corresponding linear fitting results. Inset: optical image of the Hall-bar device with its channel along the a-axis. The thicknesses of regions I-IV (from left to right) are 23 nm, 20 nm, 18 nm and 15 nm, respectively. Etching-induced sign reversal of $V_{xy}^{2\omega}$ is also observed, well consistent with the result shown in Fig. 4a.

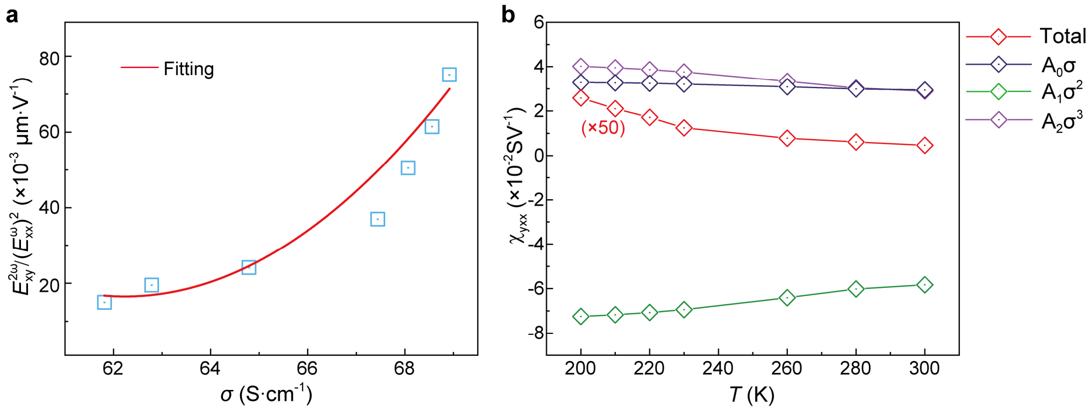

Supplementary Fig.12 | Scaling law analysis on the temperature dependent NLHE behaviors. a, $E_{xy}^{2\omega}/(E_{xx}^{\omega})^2$ as a function of σ and corresponding fitting curve using Equation (S18). **b,** Temperature dependence of χ_{yxx} and the derived three components ($A_2\sigma^3$, $A_1\sigma^2$ and $A_0\sigma$). Here the total χ_{yxx} is amplified by fifty times for better comparison. Solid lines are guides for eyes.

Supplementary references:

1. Du, Z. Z., Wang, C. M., Sun, H. P., Lu, H. Z. & Xie, X. C. Quantum theory of the nonlinear Hall effect. *Nat. Commun.* **12**, 5038 (2021).
2. Du, Z. Z., Wang, C. M., Li, S., Lu, H. Z. & Xie, X. C. Disorder-induced nonlinear Hall effect with time-reversal symmetry. *Nat. Commun.* **10**, 3047 (2019).
3. Isobe, H., Xu, S. Y. & Fu, L. High-frequency rectification via chiral Bloch electrons. *Sci. Adv.* **6**, eaay2497 (2020).
4. Duan, J. et al. Giant Second-Order Nonlinear Hall Effect in Twisted Bilayer Graphene. *Phys. Rev. Lett.* **129**, 186801 (2022).
5. Hemour, S. & Wu, K. Radio-frequency rectifier for electromagnetic energy harvesting: development path and future outlook. *Proc. IEEE* **102**, 1667-1691 (2014).
6. Auton, G., Kumar, R. K., Hill, E. & Song, A. M. Graphene triangular ballistic rectifier: fabrication and characterisation. *J. Electron. Mater.* **46**, 3942-3948 (2017).
7. Dames, C. & Chen, G. 1ω, 2ω, and 3ω methods for measurements of thermal properties. *Rev. Sci. Instrum.* **76**, 124902 (2005).
8. Ma, Q. et al. Observation of the nonlinear Hall effect under time-reversal-symmetric conditions. *Nature* **565**, 337-342 (2019).
9. Kang, K., Li, T., Sohn, E., Shan, J. & Mak, K. F. Nonlinear anomalous Hall effect in few-layer WTe_2. *Nat. Mater.* **18**, 324-328 (2019).
10. Kumar, D. et al. Room-temperature nonlinear Hall effect and wireless radiofrequency rectification in Weyl semimetal $TaIrTe_4$. *Nat. Nanotechnol.* **16**, 421-425 (2021).
11. Ho, S.-C. et al. Hall effects in artificially corrugated bilayer graphene without breaking time-reversal symmetry. *Nat. Electron.* **4**, 116-125 (2021).
12. Qin, M.-S. et al. Strain tunable Berry curvature dipole, orbital magnetization and nonlinear Hall effect in WSe_2 monolayer. *Chin. Phys. Lett.* **38**, 017301 (2021).
13. He, P. et al. Quantum frequency doubling in the topological insulator Bi_2Se_3. *Nat. Commun.* **12**, 698 (2021).
14. Min, L. et al. Strong room-temperature bulk nonlinear Hall effect in a spin-valley locked Dirac material. *Nat. Commun.* **14**, 364 (2023).
